# Changes in plasma aldosterone level after weight loss by bariatric surgery in morbidly obese patients

**DOI:** 10.1186/s12902-020-0527-4

**Published:** 2020-04-06

**Authors:** Preaw Suwannasrisuk, Patchaya Boonchaya-anant, Natnicha Houngngam, Suthep Udomsawaengsup, Sarat Sunthornyothin

**Affiliations:** 1 Division of Endocrinology and Metabolism, Department of Medicine, Faculty of Medicine, Chulalongkorn University, Bangkok, Thailand; 2 Department of Medicine, Faculty of Medicine, Naresuan University, Phitsanulok, Thailand; 3 Excellence Center in Diabetes, Hormones and Metabolism, King Chulalongkorn Memorial Hospital, Thai Red Cross Society, Bangkok, Thailand; 4 Department of Surgery, Faculty of Medicine, Chulalongkorn University, Bangkok, Thailand

**Keywords:** Plasma aldosterone concentration, Direct renin concentration, Renin–angiotensin–aldosterone system, Bariatric surgery, Obesity

## Abstract

**Background:**

Overactivation of renin-aldosterone-angiotensin system (RAS) is part of the pathogenesis of obesity-associated hypertension. Evidences have shown that weight loss can result in reduction in blood pressure and RAS. This study was aim to investigate changes of plasma aldosterone concentration (PAC) after bariatric surgery.

**Methods:**

A prospective study was done in 14 morbidly obese patients undergoing bariatric surgery. Patients who were taking medications that can interfere with PAC and renin levels were excluded. Collection of blood samples were done at baseline and at 3 and 6-month post-bariatric surgery.

**Results:**

Four out of 14 patients had hypertension at baseline. Mean body mass index (BMI) was 56.7 ± 13.5 kg/m^2^. PAC were significantly decreased at 3-and 6-month post-bariatric surgery from 14.3 ± 8.0 to 7.5 ± 5.5 [reduction of 36% from baseline (*P* < 0.01)] and 8.0 ± 6.6 ng/dl [reduction of 32% from baseline (*P* < 0.05)] respectively. The reduction of PAC at 3-month post-surgery was correlated with the reduction of body weight (*r* = 0.46, *P* < 0.05), waist circumference (*r* = 0.73, *P* < 0.05) and percent of body fat (*r* = 0.58, *P* < 0.05).

**Conclusions:**

Maximal reduction of PAC in obese patients underwent bariatric surgery occurred during the first 3 months after bariatric surgery. The reduction of PAC was associated with the reduction of body weight, waist circumference and percent of body fat suggesting the link between RAS and obesity-mediated hypertension.

## Background

The worldwide prevalence of obesity has been increasing in both developed and developing countries [1, 2]. Obesity, defined as excessive adipose tissue accumulation, contributes to many comorbidities and hypertension is one of the most common comorbidity found in 40–50% of obese patients [3]. Not only the site of energy storage, adipose tissue is also known as an endocrine organ that can secrete many adipokines and cytokines affecting the whole body [4].

Multiple mechanisms have been proposed in obesity-associated hypertension. Excess adipose tissue, adipocyte hypertrophy and dysregulation of adipokines due to chronic low grade inflammation process occur in obese condition [5]. Adipose tissue secrete some adipokine such as leptin which itself can stimulate sympathetic nervous system. Sympathetic overactivity can activate systemic renin–angiotensin–aldosterone system (RAS). Moreover, activation of local RAS by increased angiotensinogen secretion from adipose tissue has also been reported in both human and animal models [6–9]. Therefore, overactivation of both systemic and local RAS could be responsible for the pathogenesis of obesity-associated hypertension.

Previous studies have shown an improvement in blood pressure after 5% weight loss by lifestyle modification and weight loss can lead to a reduction in RAS activity in plasma and adipose tissue [10, 11]. Currently, bariatric surgery is an effective treatment for obesity. Several studies have demonstrated an improvement of blood pressure accompanied by a decrease in plasma aldosterone concentration (PAC) at 6–12 months after bariatric surgery [12, 13]. Nonetheless, early changes of aldosterone prior to 6 months after bariatric surgery while the patient is actively losing weight has not yet been investigated. In this study, we evaluated the changes of PAC at 3 and 6 months after weight loss by bariatric surgery.

## Methods

A prospective study was conducted in morbidly obese patients undergoing bariatric surgery at King Chulalongkorn Memorial hospital (KCMH), Bangkok, Thailand. Inclusion criteria were adults aged between 18 and 60 years and meeting bariatric surgery criteria by The 1991 NIH Consensus Conference [14]. Exclusion criteria were pregnancy, active cerebrovascular disease and cardiovascular disease within 6 months, chronic kidney disease more than stage 3 and history of heart failure. Symptomatic heart failure was excluded by careful history and physical examination. Patients who were taking medication that can interfere with PAC and direct renin concentration (DRC) were excluded. All subjects gave written informed consent and the study was approved by Faculty of Medicine, Chulalongkorn University’s ethical committee.

Laparoscopic sleeve gastrectomy (LSG) and laparoscopic roux-en-Y gastric bypass (LRYGB) were two standardized surgical procedures at KCMH which were chosen by a multidisciplinary team based on patient‘s BMI, comorbidities and preference. All surgeries were performed by a single surgeon. For LRYGB, the creation of a 20-to-30-ml gastric pouch, a 150-cm Roux limb, and a 50-cm biliopancreatic limb were done. For LSG, gastric-volume reduction of 75 to 80% was done by resecting the stomach alongside.

All patients were evaluated for health status, anthropometric parameters, and blood tests. All patients were fasted for 8–12 h and the collections of blood samples were done with sitting position in the early morning at baseline and at 3 and 6 month post-bariatric surgery. Radioimmunoassay is the method of blood tests for PAC and DRC. Samples were analyzed for PAC (ALDO-RIA kit; CIS bio international) and the intra-assay and inter-assay variations were 8 and 6.7, respectively and for DRC (kit; CIS bio international) and the intra-assay and inter-assay variation were 3.6 and 5 respectively. The other blood tests were analyzed by Architect C8000:Abbott at the central lab of KCMH.(sodium,potassium and chloride; ion selective electrodes (indirect), bicarbonate, creatinine, lipid profile and fasting plasma glucose; enzymatic method, HbA1c; enzymatic (photometric) method). Estimated glomerular filtration rate (eGFR) is calculated using the CKD-EPI equations. Percentage of body fat was calculated by bioelectrical impedance analysis (BIA), body weight, waist circumference and blood pressure data were collected during each visit. Body composition was measured using bioelectrical impedance analysis by Inbody 720. Body composition analyzed when the subject is fasting. Waist circumference was measured by a single investigator according to World Health Organization (WHO) steps protocol. Blood pressure was measured by automated device (Omron machine) with appropriate cuff size, blood pressure was recorded three times and the mean of second and third records were analysed. The participants rested for 3 min between each reading according to WHO steps protocol.

### Statistical analysis

Statistical analysis was performed with the SPSS version 23.0 software (SPSS, Chicago, IL). Continuous variables were described as means + SD. Differences in continuous variables between pre- and post-surgery were assessed with paired t-test.

*P* values were adjusted by Bonferroni correction for evaluation of longitudinal changes in PAC after bariatric surgery. Pearson correlation test was used to test the relationship between the two parameters. A *P* value of < 0.05 was considered statistically significant.

## Results

Nineteen patients were enrolled in the study and all participants were Asians. One patient was excluded due to active heart failure, four patients did not complete follow-up visits after surgery. As a result, fourteen patients were included for the final analyses. Baseline characteristics of participants are shown in Table 1. Four patients had hypertension prior to surgery. Their previous medications were as follow; losartan, manidipine and amlodipine. Then the medications were changed to doxazosin and/or verapamil prior to the measurement of plasma aldosterone concentration (PAC)/direct renin activity (DRC). All patients had obstructive sleep apnea. Nine patients underwent LSG and 5 had LRYGB.

**Table 1:** Baseline characteristics of the study participants [*n* = 14]

Baseline characteristics	
Age [years]	26.5 + 5.4
Sex [n[%]]	
Male	8 [57.1%]
Female	6 [42.9%]
Comorbidities [n[%]]	
Hypertension	4 [28.6%]
Diabetes	2 [14.3%]
Impaired fasting glucose	2 [14.3%]
Dyslipidemia	7 [50%]
Obstructive sleep apnea	14 [100%]
Type of surgery [n[%]]	
Roux-en-Y-gastric Bypass [LRYGB]	5 [35.7%]
Sleeve gastrectomy [LSG]	9 [64.3%]

Data are expressed as means + SD or number [percentage]

**Fig. 1:**
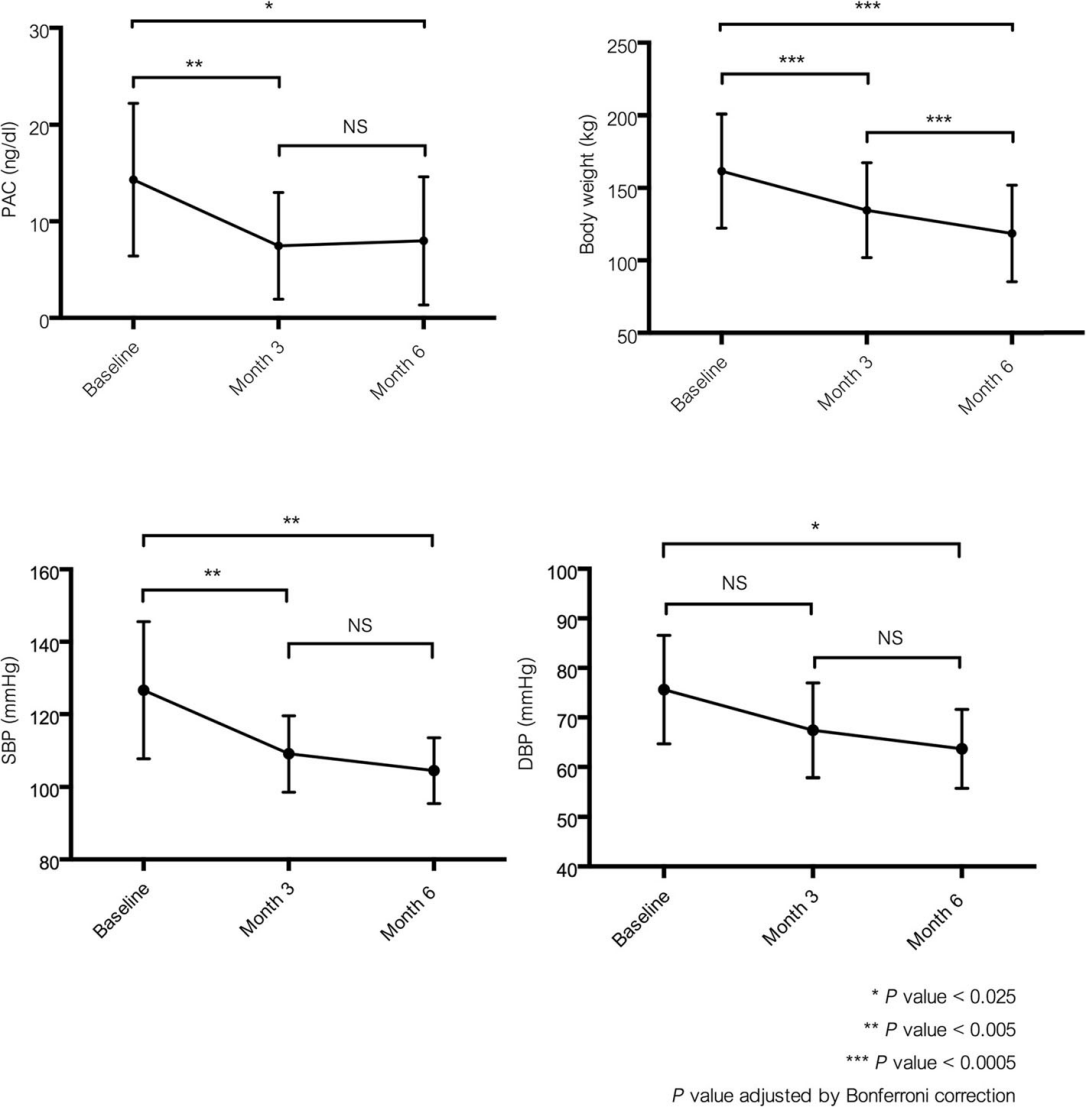
The changes of plasma aldosterone concentration [PAC], body weight and blood pressure after bariatric surgery

The changes in clinical parameters are shown in Table 2. Anthropometric measurement including BMI, body weight and waist circumference significantly decreased after bariatric surgery. Most of the patients had normal PAC levels at baseline and PAC decreased significantly at 3-month post-bariatric surgery [reduction of 36% from baseline (*P* < 0.005)] and at 6-month post-bariatric surgery [reduction of 32% from baseline (*P* < 0.025)] (Table 2 and Fig. 1). There was no significant change in PAC from 3-month to 6-month post-bariatric surgery (*P* = 0.69). No changes in DRC and potassium levels were observed both at 3-month and 6-month post-bariatric surgery. A reduction in SBP was seen at 3-month post-bariatric surgery (*P* = 0.001) whereas a reduction of DBP was seen later at 6-month post-bariatric surgery [*P* = 0.008] (Fig. 1). Fasting plasma glucose and triglyceride levels decreased significantly after bariatric surgery and weight loss.

**Table 2:** Baseline and changes in clinical and laboratory parameters at 3 and 6 months after bariatric surgery

	Baseline [*n* = 14]	Month 3 [*n* = 14]	Month 6 [*n* = 13]
BW [kg]	161.6 + 39.4	134.6 + 32.7***	118.6 + 33.4***
BMI [kg/m^2^]	56.7 + 13.5	47.1 + 11.1***	40.6 + 9.9***
Waist [cm]	153.6 + 32.2	131.5 + 25.0***	118.6 + 33.4***
Percent of fat	55.46 + 6.23	49.81 + 5.64***	–
SBP [mmHg]	126.6 + 19.0	109.1 + 10.5**	104.5 + 9.0**
DBP [mmHg]	75.7 + 11.0	67.4 + 9.6	63.7 + 7.9*
FPG [mg/dl]	97.6 + 12.6	93.3 + 23.0	83.3 + 5.9*
HbA1c [%]	5.8 + 0.6	5.5 + 0.4	5.5 + 0.4
HbA1c [mmol/mol]	40 + 1.8	37 + 1.2	37 + 1.2
Total cholesterol [mg/dl]	206.1 + 46.9	179.4 + 33.3	187.6 + 25.9
HDL-C [mg/dl]	38.9 + 12.7	32.0 + 6.0*	34.7 + 7.3
Triglyceride [mg/dl]	159.1 + 61.8	114.6 + 35.6**	102.9 + 38.5**
LDL-C [mg/dl]	133.9 + 43.4	127.6 + 28.8	135.7 + 23.1
PAC [ng/dl]	14.3 + 8.0	7.5 + 5.5**	8.0 + 6.6*
DRC [ng/L]	11.4 + 8.3	7.4 + 2.5	9.1 + 7.7
Potassium [mEq/l]	4.5 + 0.26	3.7 + 0.26	3.6 + 0.26
Creatinine [mg/dl]	0.76 + 0.16	0.70 + 0.17	0.69 + 0.15*
eGFR [ml/min/1.73m^2^]	119.4 + 16.0	126.2 + 16.3	126.9 + 15.8*

*Abbreviations*: *BW* body weight, *PAC* plasma aldosterone concentration, *DRC* direct renin concentration, *SBP* systolic blood pressure, *DBP* diastolic blood pressure, *FPG* fasting plasma glucose, *HDL- C* high – density lipoprotein cholesterol, *LDL-C* low – density lipoprotein cholesterol, *eGFR* estimated glomerular filtration rate

* *P*-value < 0.025 compared with baseline

** *P*-value < 0.005 compared with baseline

*** *P*-value < 0.0005 compared with baseline

*P*-value adjusted by Bonferroni correction

**Fig. 2:**
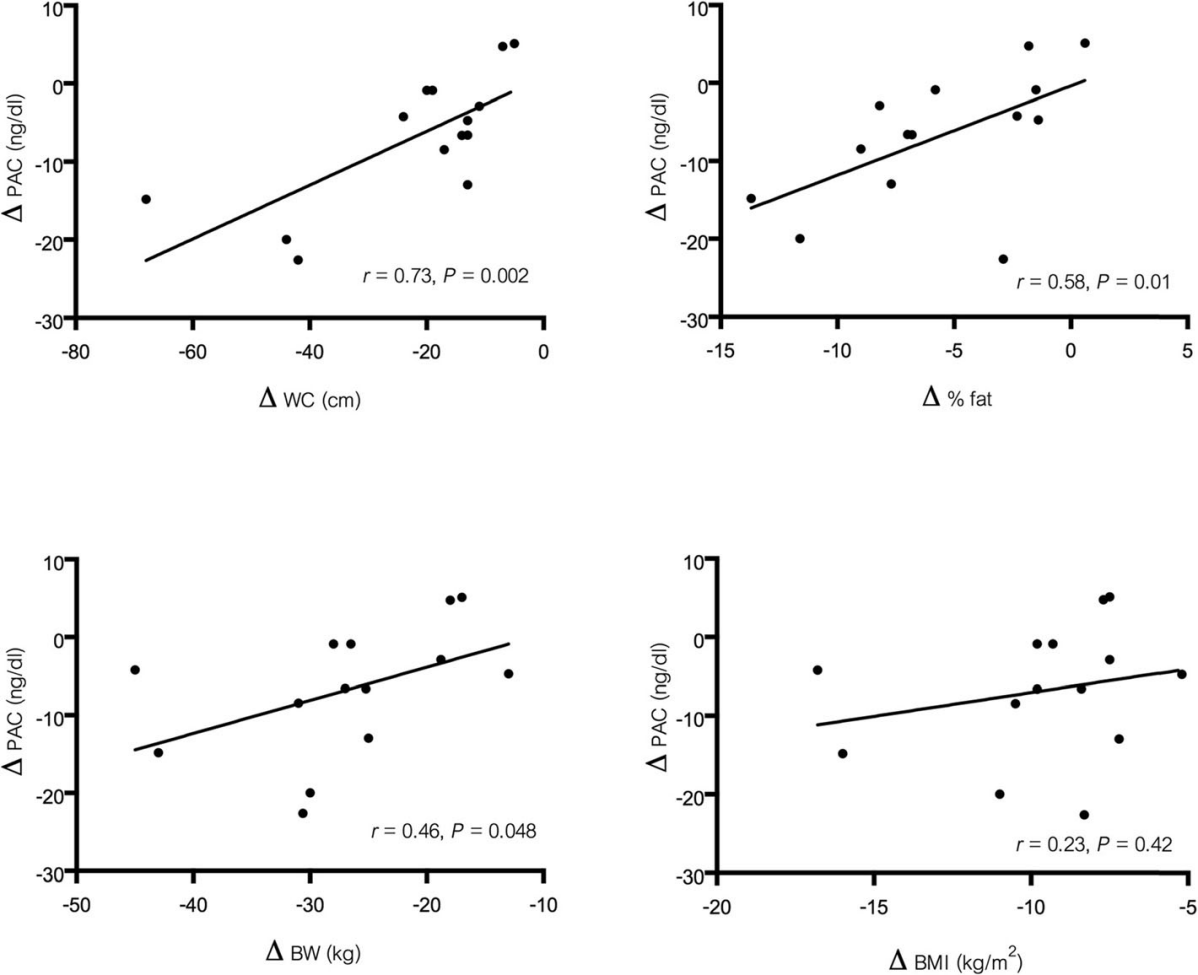
Correlations between changes in waist circumference, percent body fat, body weight, BMI and changes in plasma aldosterone concentration [PAC] at 3 months after bariatric surgery

Figure 2 shows the correlations between changes in PAC and changes in anthropometric measurement. The reduction in PAC at 3-month post-surgery was correlated with the reduction of body weight (*r* = 0.46, *P* < 0.05), waist circumference (*r* = 0.73, *P* < 0.05) and percentage of body fat (*r* = 0.58, *P* < 0.05) but not with the change in DRC. The reduction in PAC at 3-month post-surgery tended to be correlated with the reduction of BMI (*r* = 0.23, *P* = 0.42) but did not reach statistical significance.

## Discussion

We performed a prospective study evaluating early change in PAC in extremely obese patients after weight loss by bariatric surgery. We found the early reduction in PAC during the first 3 months after bariatric surgery in accompany with the early reduction in the SBP. The reduction in PAC was correlated with the reduction in body weight, waist circumference and percentage of body fat. All of the patients had significant body weight reduction and improvement in glycemic and metabolic parameters as expected by bariatric surgery.

Several studies have examined the reduction in PAC and the improvement in blood pressure at 6–12 months after bariatric surgery when the body weight loss has stabilized. A retrospective study by Ruano et al. showed the decline in PAC at 6 months after bariatric surgery and the data showed significant correlation between the reduction in PAC and BMI [12]. Dall‘s Asca et al. also reported a reduction in PAC and blood pressure by laparoscopic adjustable gastric banding after 1 year of surgery [13]. A prospective study done by Flores et al. has demonstrated that both BMI and PAC were significantly decreased at 1 year after LRYGB and LSG [15].

The novel finding in our study is that the maximum reductions in PAC and SBP occurred early at 3 months after bariatric surgery and the changes mitigated from 3 to 6 months after surgery despite the continuous decline in body weight. In our study, the reduction of aldosterone level achieved at 3 months was 7.5 ng/dl which was at the lower end of normal PAC level, thus no further reduction of PAC can be seen at 6 months. We also found that the reduction in PAC is not correlated with DRC but rather correlated with the decline in body fat mass. The reduction in PAC is perhaps a major explanation of an improvement in SBP seen early at 3 months after bariatric surgery.

Obesity-associated hypertension involves several mechanisms. The important one is the increase in both local and systemic RAS. Firstly, all component of RAS have been found in adipose tissue including angiotensinogen, angiotensin I and angiotensin II, and the increase in local adipose tissue RAS gene expression has been described in both human and animal studies [7]. Secondly, systemic RAS is also overactivated in obesity. Hyperleptinemia and obstructive sleep apnea, frequently found in obese person, can activate the sympathetic nervous system which can in turn stimulate renin release and activate systemic RAS system [16]. The reduction of leptin level after bariatric surgery has already been demonstrated in other studies [17, 18]. In our study, we also found a significant decrease in PAC but not in DRC suggesting that local RAS from adipose tissue, rather than systemic RAS, could be a major driver of elevated blood pressure seen in morbidly obese person.

Engeli et al. reported that the 5% reduction in body weight decreased plasma aldosterone level and SBP in company with the decrease in angiotensinogen gene expression in subcutaneous adipose tissue [10]. This could support our study’s finding suggesting an important role of adipose tissue RAS in obesity-associated hypertension. Further study examining changes in adipose tissue RAS gene expression after weight loss by bariatric surgery is of interest. It is still unclear what other factors may regulate adipose tissue RAS activity, however, insulin and cytokines have been proposed to mediate adipose tissue angiotensinogen production in animal models [19, 20]. The reduction in the inflammatory cytokine and the improvement in insulin sensitivity could play a role in the reduction in blood pressure after RYGB as demonstrated in the GATEWAY study [21].

The strength of our study is that we evaluated an early phase of the changes in PAC after weight loss by bariatric surgery. Our participants were mostly at a young age and all of them had an improvement in SBP suggesting that we did not include participants with essential hypertension in our study. Additionally, we have very strict exclusion criteria that we have excluded patients taking anti-hypertensive medications that can interfere with PAC or DRC levels.

However, our study has some limitations. Firstly, we have included two types of bariatric procedures in our study, LRYGB and LSG, which may have different effects on the results. The difference in the magnitude of changes in the gut hormones such as ghrelin and glucagon-like peptide-1 (GLP-1) in RYGB and SG may have an effect on blood pressure regulation as well [22]. Secondly, we did not measure other adipokines such as leptin that may have a correlation with weight loss. Thirdly, BIA was used to measure percentage of body fat which may result in underestimation of the percentage of body fat in morbidly obese patients [23, 24].

## Conclusion

Maximal reduction of PAC in obese patients underwent bariatric surgery occurred during the first 3 months after bariatric surgery. PAC did not continue to significantly decrease afterwards at 6 months despite further reduction of body weight. The reduction of PAC was associated with the reduction of body weight, waist circumference and percent of body fat suggesting the link between RAS and obesity-mediated hypertension.

## Data Availability

Data and materials are available upon request to the corresponding author.

## Abbreviations

RAS: Renin–angiotensin–aldosterone system
PAC: Plasma aldosterone concentration
DRC: Direct renin concentration
SBP: Systolic blood pressure
DBP: Diastolic blood pressure
